# Assessment of Motor Planning and Inhibition Performance in Non-Clinical Sample—Reliability and Factor Structure of the Tower of London and Go/No Go Computerized Tasks

**DOI:** 10.3390/brainsci11111420

**Published:** 2021-10-27

**Authors:** Ernest Tyburski, Magdalena Kerestey, Pavlo Kerestey, Stanisław Radoń, Shane T. Mueller

**Affiliations:** 1 Department of Health Psychology, Pomeranian Medical University in Szczecin, 71-460 Szczecin, Poland; 2 Institute of Psychology, University of Szczecin, 71-017 Szczecin, Poland; magdalena@kerestey.net; 3 ThoughtWorks, 10969 Berlin, Germany; pavlo@kerestey.net; 4 Department of Social Sciences, Pontifical University of John Paul II, 31-002 Cracow, Poland; biuro@ipri.pl; 5 Department of Cognitive and Learning Sciences, Michigan Technological University, Houghton, MI 49931, USA; shanem@mtu.edu

**Keywords:** motor planning, motor inhibition, Psychology Experiment Building Language, Tower of London, Go/No Go task, test-retest reliability, factor structure

## Abstract

In two studies, we examine the test-retest reliability and factor structure of the computerized Tower of London (TOL) and Go/No Go (GNG). Before analyses, raw results of variables that were not normally distributed were transformed. Study 1 examined the reliability of a broad spectrum of indicators (Initial Time Thinking, ITT; Execution Time, ET; Full Time, FT; Extra Moves, EM; No Go Errors, NGE; Reaction Time for Go Responses, RTGR) across an eight-week delay in a sample of 20 young adults. After correction for multiple comparisons and correlations, our results demonstrate that the tasks have ambiguous test-retest reliability coefficients (non-significant *r* for all indicators, and interclass correlation (ICC) for TOL; significant ICC for GNG; show lack of reliable change over time for all indicators in both tasks); moreover, ITT exhibits strong practice effects. Study 2 investigated both tasks’ factor structure and conducted a more detailed analysis of indicators for each trial (ITT, ET, EM) in the TOL task in the group of 95 young adults. Results reveal a satisfactory 2-factor solution, with the first factor (planning inhibition) defined by ITT, NGE, and RTGR, and the second factor (move efficiency) defined by EM and ET. The detailed analysis identified a 6-factor solution with the first factor defined by ITT for more difficult trials and the remaining five factors defined by EM and ET for each trial, reflecting move efficiency for each trial separately.

## 1. General Introduction

Adequate measurement of executive functions (EFs) in healthy and clinical individuals is still a matter of contention. According to Chan et al. [1], EFs are an umbrella term comprising a wide range of cognitive processes and behavioral competencies. They generally refer to higher-level cognitive functions involved in controlling and regulating lower-level cognitive processes and goal-directed, future-oriented behavior [2].

Two important aspects of EFs are planning and inhibition. Planning is the ability to identify and organize the steps toward a particular goal [3]. It is commonly measured by disk-transfer tests such as the Tower of London task (TOL) [4]. The different versions of the TOL task and the resulting differences in structural properties, problem space, measures, and administration lead to difficulty in reaching conclusions regarding its psychometric properties. It is unclear whether different versions of the task measure the same components of EFs [5,6]. For example, some versions of the task require the participant to find the shortest number of steps to solve the task and assess the time needed to plan and execute the task perfectly; other versions permit mistakes and measure the time and number of steps needed to solve problems. TOL problem difficulty is usually defined only in terms of the minimum number of moves required for an efficient solution; infrequently, the number of indirect moves and number of optimal paths to a solution is used. Kaller et al. [7] suggested that level of difficulty can be more accurately characterized by a depth of search, defined as the number of intermediate moves before the first ball/disc can be placed in the goal position. Similarly, the difficulty may be indicated by the goal hierarchy, which is related to the degree to which the sequence of final goal moves can be derived from the configuration of the goal state [7,8]. This approach requires the use of iso-problems (the same problem with differently colored balls/discs) for each measurement over time, which raises the question of how equivalent the iso-problems indeed are [9].

Inhibition is the ability to effortfully suppress responses that are inappropriate for the current task. It depends greatly on what is being inhibited; therefore, it is a highly complex executive function that comprises diverse components. One main distinction is between motor inhibition, where prepotent motor reactions are inhibited, and interference inhibition, where the processing of irrelevant stimulus characteristics has to be inhibited [10]. Motor inhibition can be further considered in terms of reactive inhibition, defined as the ability to stop response immediately at the presentation of stop instruction, and proactive inhibition, defined as the ability to modify the motor strategy according to the context [11]. Because TOL involves motor functions, we suspected that motor inhibition might play an important role in it. Therefore, we choose GNG from representative psychological tasks used to assess inhibitory control advised by Diamond [12] and Nigg [13]. It is worth noticing that GNG measures reactive inhibition only in one motor aspect–action restraint. It does not measure action cancelation as the stop-signal task does [14]. The basic goal for participants in Go/No Go tasks is to respond to one stimulus category (Go stimuli) and inhibit responses to another category (No Go stimuli) [15]. Versions of the GNG task differ mostly in terms of the stimuli presented to the participant, which can be visual (pictures, words, letters) or auditory, and the required reaction, which, in most cases, is a motor response. Some authors suggest that inhibition and planning are related hierarchically, with inhibition being a basic process, and planning being more advanced, requiring more complex mental operations [12]. While there is no universal agreement on EF’s structure, researchers often try to verify it using factor analyses.

One of the main problems with measuring EFs is the novelty of stimuli: measures of these processes can never be completely reliable because they are designed to assess the ability to cope with new problems, but problems cease to be novel when a test is readministered [16]. One can counteract this problem by using an alternative version of the task, but this is rarely the case in EFs measurements. Although equivalent versions of the task do not entirely eliminate practice effects, they can help limit them, allowing for more accurate measurement of EFs over shorter periods. It has been suggested that practice effects can occur even after six months [17]. Therefore, reliability assessment in the EF tasks should assume and control for practice effect.

Literature on reliability of TOL and GNG tasks and their validity shows various shortcomings, which encouraged us to conduct present research. Among popular and free Psychology Experiment Building Language (PEBL) battery, we selected a TOL version constructed for healthy adults to avoid the common problem of ceiling effect. Inhibition is an important executive function involved in TOL apart from planning, we therefore decided to use the visual GNG task to measure adequate type of motor inhibition. Chosen versions for both TOL and GNG have alternative versions for each assessment over time which help to decrease practice effects. This investigation has two main aims, addressed in two separate studies using two distinct data sets acquired from healthy young adults. The first aim (Study 1) examines the test-retest reliability of the TOL and GNG with the control practice effects. The second aim (Study 2) examines the factorial structure of TOL and GNG. Results presented in both studies are part of the broader research.

## 2. Study 1

### 2.1. Introduction

The test-retest reliability of different indicators of TOL and GNG has been studied by a few past researchers, for a variety of versions of those tasks. A survey of studies, presented in Table 1 for TOL and Table 2 for GNG, revealed that most studies report only Pearson’s *r* correlation, which is considered a weak measure of test-retest reliability because it is only a measure of correlation. Therefore, some authors also used Intraclass Correlation (ICC), which better measures this type of reliability because it reflects both degrees of correlation and agreement between measurements [18]. Furthermore, the survey revealed that, most of the studies investigated a group of students over a short period without using an alternative version of the tests in a second measurement. Thus, research provides an ambiguous set of results due to different populations involved and different intervals used.

Current study extends the findings of Piper et al. [26] regarding the test-retest reliability of executive functions’ measures from the PEBL battery. In contrast to Piper’s research [26], we investigated the reliability of more indicators of TOL and GNG in a sample of healthy young adults (20–40 y.o.) over a 2-month interval, and more complex statistical analysis. The classical version of TOL proposed by Shallice [31] is a simple task for healthy adults; therefore, it is prone to a ceiling effect in the second measurement. To counteract this problem, we used the version of the TOL task, which has a more complicated problem space. In the second measurement, we used an alternative version of the task with different trials and the same level of difficulty. In contrast to previous research, we analyzed a broader spectrum of indicators in this study: Initial Time Thinking (ITT), Execution Time (ET), Full Time (FT), Extra Moves (EM), No Go Errors (NGE), and Reaction Time for Go Responses (RTGR). In addition to the simple correlation and ICC statistics reported in most studies, we have employed other methods for measuring stability over time, such as Reliable Change Index (RCI), which provides a more precise estimate of relative change and controls for test reliability [17]. Just like Köstering et al. [23], we performed ANOVA analyses for each level of difficulty in the TOL task in order to investigate changes over time more precisely.

### 2.2. Methods

#### 2.2.1. Participants

Thirty-five young adults participated in the study. They were recruited via the university website, posters, social networks, local radio, and adverts in newspapers. There was no financial compensation for participation. All participants met the following inclusion criteria: (1) being a native Polish speaker; (2) being between 20 and 40 years old; and (3) having normal or corrected-to-normal vision and hearing. Participants with (1) a history of neurological disorder; (2) any psychiatric disorder; (3) a history of drug addiction; (4) any head injury; or (5) education in psychology were excluded from data analysis using a self-report screening questionnaire.

Additionally, we used the General Health Questionnaire 30 (GHQ-30) to assess mental health problems. Crystallized, and fluid intelligence was measured using the Information, and Picture Completion tests from the WAIS-R battery, respectively as proposed by Lezak (1995). One participant was excluded from the analysis due to an extreme result greater than 1 *SD* from the mean on the GHQ (the cut-off points are 99.13 for people aged less than 30, and 95.69 for people aged 30–40 [32]). No participants were excluded due to results on intelligence tests. One participant was excluded due to missing data.

Twenty of the participants took part in both assessments; the rest participated only in the first. Analyses were restricted to participants who completed both assessments. The final sample consisted of 14 women and 6 men, aged 21–40 years old (*M* = 29.95; *SD* = 6.25), with 15–23 years of education (*M* = 18.63; *SD* = 2.42), raw scores on GHQ-30 from 44 to 87 (*M* = 60.55; *SD* = 10.91), WAIS-R raw scores for Information from 8 to 22 (*M* = 15.80; *SD* = 3.61) and Picture Completion from 15 to 34 (*M* = 26.30; *SD* = 4.76).

The Ethics Board of the Institute of Psychology of the University of Szczecin approved the research procedure (KB 9/2018). During the first assessment, each participant gave informed consent and completed computerized versions of the Tower of London (TOL) and Go/No-Go (GNG) tasks from the Psychology Experiment Building Language (PEBL). The tests were administered and scored following the standard procedures by a group of six well-trained examiners under the leading investigators’ supervision. The testing took place in a quiet setting at the University. After eight weeks, participants were invited again and tested with an alternative version of the same tasks in the same order.

#### 2.2.2. Tasks and Measurements

We used computerized versions of the TOL and GNG tasks from the PEBL. The PEBL is open-source software, licensed under the GNU General Public License 2.0, which allows scientists to create and conduct neuropsychological tests, primarily devoted to experimental design [33]. Instructions for each task were translated from English into Polish and then evaluated by two expert judges whose advice was carefully considered and applied when relevant.

**Figure 1:**
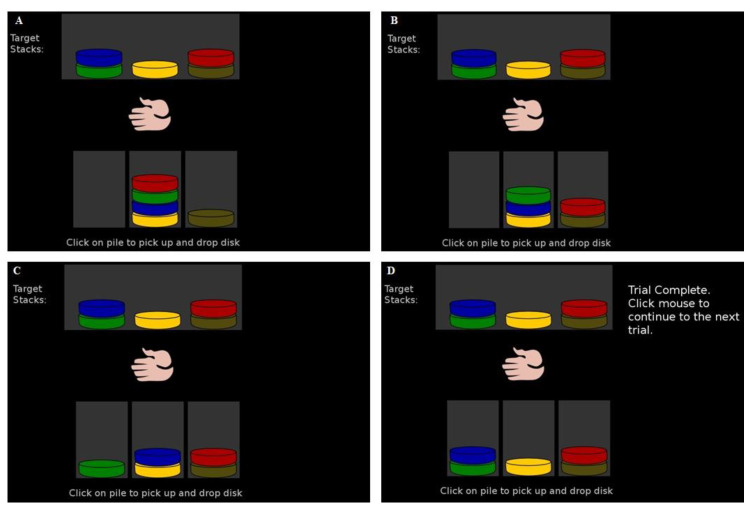
Example of a trial with the step-by-step optimal solution: (**A**) beginning state, (**B**) first move, (**C**) second move, and (**D**) third and final move.

**Figure 2:**
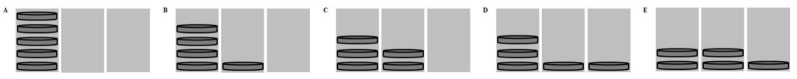
All possible kinds of configurations for the five-disc Tower of London task.

This study used Ward and Allport’s TOL task [34], adapted by Phillips et al. [35] in research on a group of non-clinical adults. The task consisted of five colored discs that can be moved, one by one, on and off three pegs of equal height. Each trial’s goal was to move all disks from an initial state to a goal state, which was also shown on the screen. All five disks can be stored on any one of the pegs. As shown in Figure 1, the color patterns of the beginning and the goal state determine a minimal number of moves needed to solve the problem in the given trial. Responses were made using a computer mouse. The computerized version of the task prevents participants from making illegal moves, eliminating one of the sources of variability previously discussed. Participants were instructed to solve the task as quickly as possible with the minimum number of moves. At the beginning of the task, we added three practice trials with three disks (no indirect moves) to ensure that participants understood the task. The main task contained eight trials with five discs. For each trial, the level of difficulty increased with the number of moves needed to solve it (3–10) and the number of indirect, counter-intuitive moves, which do not immediately bring the configuration closer to the goal state (0–6). The numbers of distinct optimal solutions remained the same for each trial (only one solution; see Figure 2, Table 3). The number of moves for the optimal solutions in Phillips version was the same on the test and retest versions for 5 of 8 trials; therefore, we analyzed only trials 1, 2, 3, 6, and 7. All participants saw the same sequence of problems on each test administration, but the problems differed across the two testing sessions. The beginning and goal states differed for each trial, but task parameters and difficulty remained the same. We measured total time spent planning, i.e., total time until the first move ITT, total execution time, i.e., the total time from the first move to completion ET, the total time for completion of all trials FT, and the total number of EM, i.e., moves made minus 31 EM. Additionally, ITT and ET were analyzed individually for each trial.

As in the GNG study of Bezdjian et al. [15], participants were requested to follow a sequential presentation of letters and respond to the target letter (P) by pressing a button on the keyboard while withholding responses to the non-target letter (R). Letters were randomly generated and presented for 500 milliseconds in one of four squares, arranged in a 2 × 2 pattern with one star in each square. The interval between stimuli lasts 1500 milliseconds. The task consisted of 160 trials with a ratio of targets to non-targets of 80:20 (128:32). At the beginning of the task, a short practice session was administered to ensure that participants understood the instructions. Because of the random generation of letters, test and retest versions differed in terms of letter sequence. Results were assessed by calculating NGE and RTGR.

#### 2.2.3. Statistical Analyses

Statistical analysis of the data was conducted using the IBM SPSS 25 Statistical package. We used the Box-Cox transformation for the EM and NGE to achieve the normality of the distribution for all analyzed variables [36]. We used paired *t*-tests to examine differences between baseline and retest performances with 1000 bias-corrected bootstrap samples. The size of the practice effects was assessed with Cohen’s *d*. We used Pearson’s *r* correlation with 1000 bias-corrected bootstrap samples. Since Pearson’s *r* correlation is considered a weak measure of test-retest reliability, especially when group means are similar and coefficients are high [37], we also used the ICC, which measures correlations within a class of data rather than correlations between two different classes of data [38]. Here we used ICC(3,1): a two-way mixed model of ICC (consistency), with a 95% confidence interval [18,38]. For multiple correlations and comparisons, we used Holm-Bonferroni corrections [39]. We also calculated Reliable Change Indices to assess whether a change between repeated assessments exceeds the probable range of measurement error (RCI) [40]. The RCI estimates the probability that a given difference in a score is not due to measurement error, but reflects real results [41]. We used RCI adjusted for controlling practice effects computed by formula [42]:

(1) $$RCI=\frac{\left(T_{2}-T_{1}\right)-\left(M_{2}-M_{1}\right)}{SED_{I}}$$

where *T*_1_—score at first assessment, *T*_2_—score at second assessment, *M*_1_—mean at first assessment, *M*_2_—mean at second assessment, *SED_I_*—standard error of the difference by Iverson.

We used alternative calculation for SED made by Iverson [43]:

(2) $$SED_{I}=\sqrt{{\left(S_{1}\sqrt{1-r_{12}}\right)}^{2}+{\left(S_{2}\sqrt{1-r_{12}}\right)}^{2}}$$

where *S*_1_—standard deviation at first assessment, *S*_2_—standard deviation at second assessment, *r*_12_—correlation coefficient between first and second assessment.

Additionally, repeated measures analysis of variance (RM-ANOVA) with the within-subject factors “Time” (Times 1 and 2) and “Trial” (Trials 1, 2, 3, 6, and 7 of TOL) were separately performed with time until the first move, and move time as the dependent variables. EM was excluded from this analysis because distribution did not reach normality, relevant skewness, and kurtosis even with transformation. We used the Bonferroni post-hoc test for both variables, and in the case of ET, we used Greenhouse-Geisser correction for degrees of freedom.

### 2.3. Results

Descriptive statistics, Student’s *t*-test, and Cohen’s *d* for all indicators on TOL and GNG tasks are shown in Table 4. After Holm-Bonferroni’s *p*-value corrections for multiple comparisons, the difference between the first and second time point was significant only for ITT of TOL, and the effect was very large.

There were no significant differences between time points for indicators in GNG. Person’s *r*, ICC, and RCI for all TOL and GNG indicators are shown in Table 5. After Holm-Bonferroni’s *p*-value corrections for multiple correlations, there were no significant Pearson’s correlations for all indicators of TOL and GNG. ICC correlations did not appear to be significant for TOL indicators but were significant for both NGE and RTGR of GNG. For ET, FT, and EM, only 5% of participants fell outside of the RCI confidence intervals, indicating reliable change overtime only for one person in the group. Interestingly, for ITT, scores did not change reliably for all of the participants. Similarly, in the GNG task, for RTGR, scores for only one person change reliably across two assessments, while for NGE, there was no reliable change over time for all participants.

**Figure 3:**
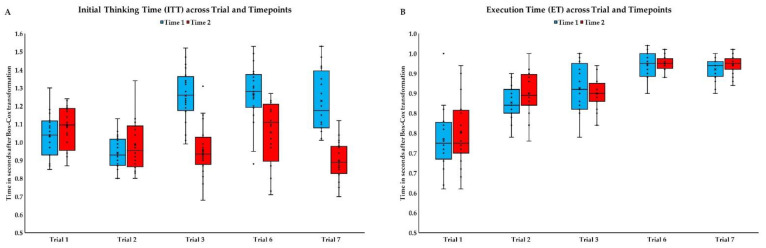
Performance in Tower of London (TOL) task, across repeated measurements: (**A**) Initial Thinking Time and (**B**) Execution Time (blue boxes, Time point 1; red boxes, Time point 2). In all box plots, the bottom end of the box designates the first quartile, a line within the box indicates the median, and the top end of the box shows the third quartile. Whiskers indicate values 1.5 times the interquartile range below the first quartile and above the third quartile. Crosses represent average values. Circles designate individual observations.

Repeated Measures-ANOVAs were performed separately for each variable from TOL (Figure 3A,B) to examine the effects of within-session trials and testing sessions For ITT, differences between both sessions (*F*_(1, 19)_ = 55.95; *p* < 0.001; ɳ^2^ = 0.75) and trials (*F*_(4, 76)_ = 18.34; *p* < 0.001; ɳ^2^ = 0.49) were significant. There were significant differences between the second trial and remaining trials (0.001 > *p* < 0.003), the first and the sixth trials (*p* = 0.004), and the sixth and seventh (*p* = 0.038). Also interaction between trail and session was significant (*F*_(4, 76)_ = 36.19; *p* < 0.001; 311 ɳ^2^ = 0.66). Comparisons revealed that the first and the second session did not differ in first and second trial, while there were significant difference for other trials. The greatest difference occurred in the seventh (difference in estimated marginal *Ms* = −0.33), the difference was smaller for the third trial (*Ms* = −0.30), and for sixth trial was the smallest (*Ms* = −0.21).

For ET, differences between trials were significant (*F*_(2.12, 40.26)_ = 105.49; *p* < 0.001; ɳ^2^ = 0.85), but the differences between sessions were not (*F*_(1, 19)_ = 1.11; *p* = 0.305; ɳ^2^ = 0.06). Significant differences occurred between the the first and remaining trials (*p* < 0.001), the second and the sixth, and the seventh trials (*p* < 0.001), the third and the sixth, and the seventh trials (*p* < 0.001). The interaction between trail and session was nonsignificant (*F*_(2.43, 46.09)_ = 1.34; 320 *p* = 0.273; ɳ^2^ = 0.07).

### 2.4. Discussion

After the correction for multiple correlations (Pearson’s *r* and ICC), results ceased to be significant for all TOL task indicators. After the correction for multiple correlations, Pearson’s *r* ceased to be significant for both GNG indicators. Both NGE and RTGR yielded a fair level of ICC.

Novelty effects and practice effects are essential factors that should be considered when testing the executive functions, especially when taking repeated measurements over time. These effects are intertwined, but the literature is equivocal about their relationship [17]. We observed a practice effect for ITT—times were significantly shorter at the second time point, and the effect size was very large [44]. For FT, ET, and EM for TOL, and both GNG indicators, Student’s t-test showed no significant differences between the two time points, suggesting the absence of practice effects. However, when controlled for practice effects, results showed that all participants fell within the 95% confidence interval for all TOL indicators, with a cut-off point of ±1.96, which indicates a lack of reliable change over time [17,40]. As with the TOL task, for both GNG measures, almost all participants fall into a 95% interval, which indicates no reliable change over time. In contrast to Köstering et al., research [23], our results suggest that both tests have ambiguous test-retest reliability coefficients. In our view, the reason for this may be transformations of skewed raw variables (Box-Cox, recommended by Sakia [36]), and corrections for multiple comparisons and correlations [39] that we applied in our study.

With RM-ANOVA, we discovered more complex practice effects for the TOL task depending on the trial’s difficulty level. A significant interaction effect for ITT suggests differentiated practice effects based on the difficulty level of the task (number of necessary direct moves or/and number of indirect moves; see Table 3). For easy first and second trials, the practice effect was absent, whereas it occurred for other, more complicated trials. Longer ITT for more difficult trials (3, 6, and 7) than easier ones (1 and 2) at the first time point, suggests that subjects developed more complex strategies for solving these problems. The lack of difference between the last three trials (3, 6, and 7), despite increasing number of indirect moves, may suggest that the identified strategies were retained (see: Figure 3A and Tables S1 and S2). Contrary to the first time point, in the second time point, despite the growing difficulty, ITT was relatively stable over trials, which may suggest the emergence of practice effects for the applied strategies.

For the ET, the effect of interaction and the main effect of time are insignificant, which suggests a lack of practice effects for this measurement. The significant main effect of the trial shows that more time was taken on the later trials due to the increasing number of movements needed for a solution (click and drag).

According to Diamond’s model [12], the fact that practice effects occur for planning (TOL), and not for motor inhibition (GNG) may result from the hierarchical structure of executive functions. Lower level executive functions like inhibition involve a lesser degree of complex cognitive operations, e.g., forming strategies for solving tasks. Higher-level executive functions, like planning, are based on this kind of operation, and therefore are more susceptible to practice effects. According to Duff [17], practice effects are stronger in tasks based on fluid abilities, where answers can be obtained in the setting, and where responses have not been met previously.

For such simple tasks like GNG used in this study, practice effects rarely occur. It would be worth verifying if the absence of practice effect would also be observed for more complex tasks which involve different stimuli (e.g., facial expression or semantic meaning [45,46,47]).

## 3. Study 2

### 3.1. Introduction

The factor structure of different tasks capturing various aspects of EF, including planning measured by TOL task and motor inhibition measured by GNG, has been studied in past research. Levin et al. [48] investigated the structure of executive functions in head-injured children using seven tests, including the Shalice version of the TOL task and the GNG task. Indicators of TOL and GNG were loaded in three following factors: planning as planning-execution dimension (TOL: percentage solved, three trials; and the number of broken rules), schema as a mental representation of the task (TOL: percentage solved, trial 1), and inhibition (TOL: initial thinking time, and GNG false alarms). Culbertson and Zillmer [49], in the group of children with ADHD, found that among different cognitive tests, all indicators in TOLDX (total move score, total time violation, total rule violation) fall under a single factor named executive planning/inhibition. Berg et al. [8] obtained three factors in their exploratory analysis for different measures of TOL task in the group of students more and less experienced with the TOL task. The first factor, labelled move efficiency, was influenced mostly by the proportion of perfect solutions, optimal move score, and the number of extra moves, and also to some degree by total solution time. The second factor, labelled solution speed, was loaded by average time per move during the solution, and total solution time. The third factor, identified as planning speed, was influenced by a single initial planning time measure.

Georgiou et al. [50] investigated the structure of planning functions in students’ groups, using a computerized version of the TOL task and different planning tasks. Indicators of TOL were loaded in both obtained factors: action planning (total number correct) and operation planning (total number correct and initial time thinking). Miyake et al. [51], in the college students group, confirmed a three-factor model with inhibition, shifting, and updating for several tasks measuring simple executive functions. Inhibition contributed to performance on the Tower of Hanoi (TOH), as indicated by the total number of moves. Bender et al. [52] in research on different response selection and response inhibition measures in a student group, confirmed a two-factor model in which GNG (errors of commission) was part of the response inhibition factor.

There is little research on executive functions’ factor structure, including TOL and GNG tasks. In contrast to previous studies, we analyzed a broader spectrum of indicators in this study: ITT, ET, EM, NGE, and RTGR. Similarly, like in the first study, we used a version of the TOL task with more complicated problem space, which counteracts the ceiling effect. Additionally, we perform more detailed factor structure and ANOVA analysis’ for each level of difficulty in the TOL task, which to our knowledge, has not been studied in previous research (for all peer-reviewed publications using the PEBL see: [26]).

### 3.2. Methods

#### 3.2.1. Participants

One hundred and seven young adults participated in the second study. Recruitment and inclusion criteria follow the same principle as study 1. Similar to study 1, we used GHQ-30 to assess mental health problems, and Information and Picture Completion tests from the WAIS-R battery to assess crystallized and fluid intelligence. Three participants were excluded from the analysis due to a result greater than 1 *SD* from the norm on the GHQ (the cut-off points are 99.13 for people aged less than 30 and 95.69 for people aged 30–40 [32]). Two participants were excluded due to results in the intelligence tests. Seven participants were excluded due to missing data.

The final sample consisted of 62 women and 33 men, aged 21–40 years old (*M* = 29.91; *SD* = 5.62), with 10–24 years of education (*M* = 17.87; *SD* = 2.81), raw scores on GHQ-30 from 38 to 98 (*M* = 63.42; *SD* = 12.74), WAIS-R raw scores for Information from 8 to 26 (*M* = 15.71; *SD* = 3.42), and Picture Completion from 15 to 36 (*M* = 26.48; *SD* = 4.17).

The Ethics Board of the Institute of Psychology of the University of Szczecin approved the research procedure, which followed the procedure from study 1. In study 2, participants took part only in single testing.

#### 3.2.2. Tasks and Measurements

We used computerized versions of the TOL and GNG tasks from the PEBL. The description of both tasks is presented in Study 1. To make studies 1 and 2 comparable, we again analyzed only trials 1, 2, 3, 6, and 7 from the TOL task (for explanation see: point 2.2.2).

#### 3.2.3. Statistical Analyses

Statistical analysis of the data was conducted using the IBM SPSS 25 Statistical package. Prior to the analyses, we used Box-Cox transformation for all variables to achieve the normality of the distribution [36]. For the investigation of the factor structure of both tasks, we used three TOL scores (ITT, ET, and EM) and two GNG scores (NGE and RTGR). Additionally, we performed detailed factor analyses for scores on each of five TOL trials (trials 1, 2, 3, 6, and 7) for ITT, ET, and EM. In both cases, we used principal components analysis with VARIMAX rotation. Factors with eigenvalues >1 (the Kaiser-Guttman criterion and scree plot) were retained, and factor loadings of 0.40 or greater were considered significant. For a more in-depth investigation of differences between trials, analysis of variance (ANOVA) was performed. We used pairwise comparison with Bonferroni correction and Greenhouse-Geisser correction for degrees of freedom.

### 3.3. Results

Descriptive statistics for performance in the first time point on TOL and GNG tasks are shown in Table 6. Exploratory factor analysis for both tasks showed a 2-factor solution (see scree plot: Figure S1), which explained 66.19% of the total variance. Rotated factor loading estimates are shown in Table 7. Factor 1, which accounted for 34.93% of the variance, was defined by ITT from TOL, NGE, and RTGR from GNG and labelled planning/inhibition. Factor 2, which accounted for 31.26% of the variance, was defined by EM and ET, and was labelled move efficiency.

Exploratory factor analysis for five TOL trials showed a 6-factor solution (see scree plot: Figure S2), which explained 77.00% of the total variance. Rotated factor loading estimates are shown in Table 8. Factor 1, which accounted for 16.34% of the variance, was defined by ITT for trials third, sixth, seventh, and was labelled strategic planning. Despite equivocal factor loading estimates, the rest of the following factors refer to separate trials. Factor 2, which accounted for 14.55% of the variance, was defined by EM and ET for the first trial and ITT for the second trial. Factor 3, which accounted for 13.27% of the variance, was defined by EM for the second trial, ITT for the first trial, and ET for the second and seventh trials. Factor 4, which accounted for 12.61% of the variance, was defined by EM and ET for the third trial. Factor 5, which accounted for 11.58% of the variance, was defined by EM and ET for the sixth trial. Factor 6, which accounted for 8.65% of the variance was defined by EM in the seventh trial ET.

**Figure 4:**
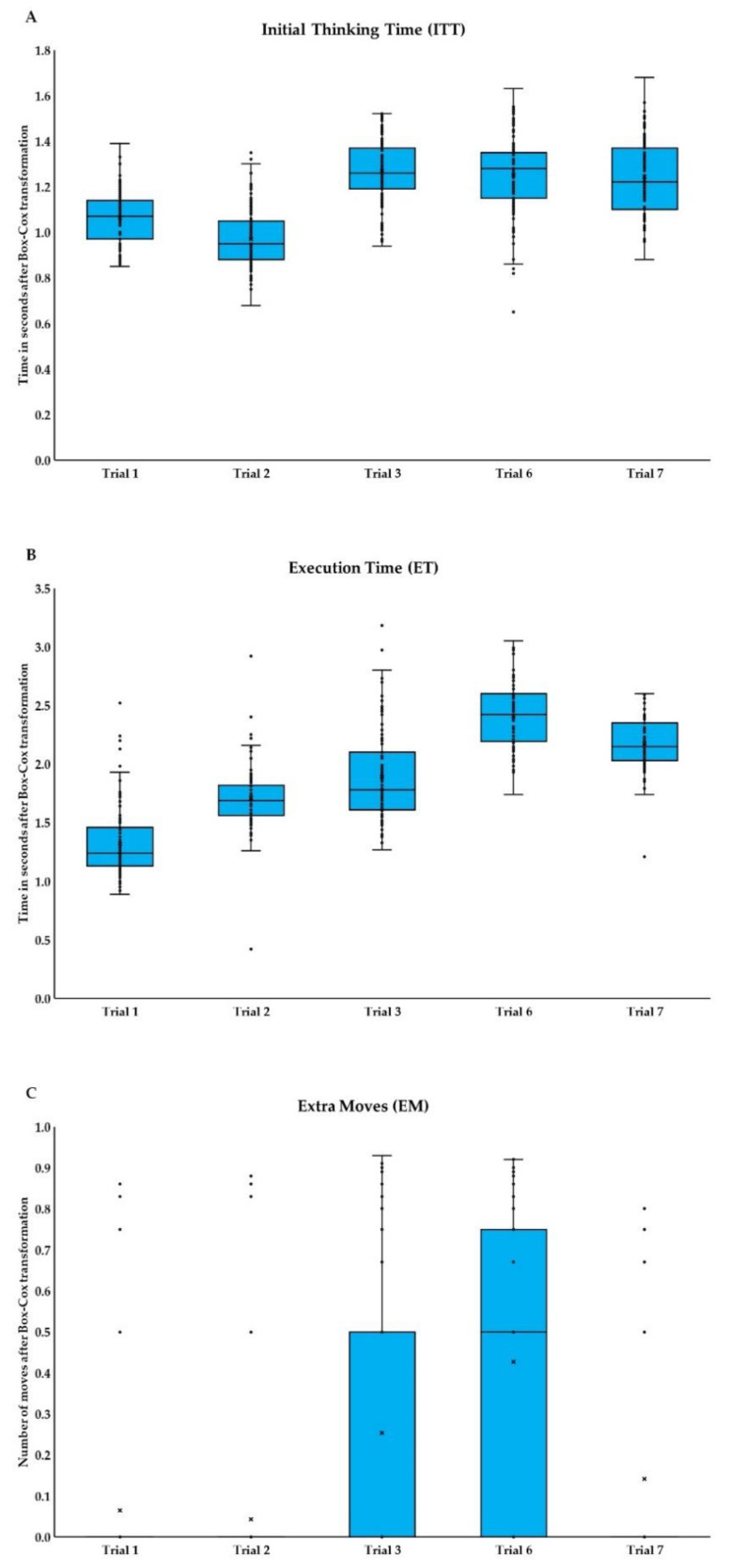
Performance in Tower of London (TOL) task in five trials: (**A**) Initial Thinking Time, (**B**) Execution Time, and (**C**) Extra Moves. Boxplots conventions as in Figure 3.

Next, ANOVAs were performed separately for each variable from TOL (Figure 4A–C, and see Table S4) to examine the differences between trials. For ITT differences between trials were significant, *F*_(3.08,289.39)_ = 114.39; *p* < 0.001; ɳ^2^ = 0.55. Differences occurred between first and all other trials (*p* < 0.001); second and all other trials (*p* < 0.001). For ET differences between trials were significant, *F*_(3.21,301.91)_ = 244.06; *p* < 0.001; ɳ^2^ = 0.72. Significant differences appeared between each trail and the rest of the trials (*p* < 0.001). Also for EM differences between trials were significant, *F*_(3.38,317.85)_ = 31.12; *p* < 0.001; ɳ^2^ = 0.25. Significant differences occurred between first and third (*p* < 0.001), and sixth trial (*p* < 0.001); second and third (*p* < 0.001), sixth (*p* < 0.001), and seventh trial (*p* = 0.022); third and sixth (*p* = 0.005); sixth with seventh (*p* < 0.001).

### 3.4. Discussion

The factor structure obtained for TOL and GNG measures had two factors that reflected planning/inhibition and move efficiency. The first factor grouped both GNG indicators, as well as ITT for TOL. Variable loadings were in opposite directions for RTGR and NGE, suggesting the occurrence of the speed-accuracy trade-off for the GNG task, which is a common effect in the tasks performed under time pressure [53]. Initial thinking time is a measure of time taken to plan all or part of the solution and can indicate both more thorough planning and ineffective planning [5]. Due to its shared variability with measures classically interpreted as indices of inhibition, we labelled first-factor planning/inhibition. Because of the correlation between ITT and ET, and lack of correlation between ITT and EM (see: Table S3), in our view, longer time of planning could result either due to the problems with creating a plan [54], or usage of more perceptual (simply making a next move that will bring the current state perceptually closer to the goal state), than goal-recursion strategy (extensive goal management and setting up a series of subgoals to achieve the superordinate goal [51]). The second factor, which included EM and ET, represented the cognitive dimension that appeared to be relevant only for the TOL task and was labelled move efficiency. According to Berg and Byrd [5], extra moves measure the solution’s efficiency, while execution time measures the speed of the solution and combines motor time taken for moving disks and cognitive time taken for additional on-line planning and error correction. In the version of the TOL task used in our research, correction of the errors required making additional moves; therefore, in our view, execution time was growing with the number of extra moves due to both motor time and cognitive time. In our study, different TOL measures represent different sources of shared variability, suggesting that they capture different executive functions [48]. Other researchers also found different factors for TOL and GNG [48,49,50], but direct comparison is limited due to differences in used versions of tasks, number of considered tasks, number of indicators for each task, and the type of sample.

More detailed factor structure analysis for different measures on each level of difficulty in the TOL task reveals a six-factor solution. The first factor is influenced by ITT for three more difficult trials and represents strategic planning. For the first two trials, with no counter-intuitive moves involved, there is no need for a longer planning period before moving the first disc. Conversely, in more difficult trials, one needs more thorough planning at the beginning. Results of ANOVA also confirmed the difference between easier trials (first and second) and more difficult trials (third, sixth, and seventh) in terms of planning time. Besides the first, all remaining factors approximately capture ET and EM for each trial separately and can be interpreted as move efficiency for each level of difficulty. Results of ANOVA corroborate differences between all trials in terms of move efficiency. Our findings in both analyses show that strategic planning and moves efficiency display themselves in diversified ways depending on the level of trial difficulty (minimal number moves to the solution number and number of counter-intuitive moves [5]).

## 4. Limitations

It is essential to view these results in the context of their limitations. Future research should investigate other types of inhibition, such as proactive inhibition or other types of motor inhibition [13]. Although the research sample was not composed of students alone, rather young adults aged 20–40, results should not be generalized to older people, as much research shows that EFs decrease with age [2]. Further research should examine the test-retest reliability and factor structure of the TOL and GNG tasks in older people, especially those in late adulthood. Present study concerns healthy individuals; therefore, results should not be directly generalized to clinical populations. There is a need for further investigation of this version of TOL and GNG tasks in clinical samples, where test-retest reliability depends on a much greater number of factors (disease progression, fluctuation of neurological and psychiatric symptoms, and the treatments being used [17]) and where factor structure may be somewhat different [55]. As with other research on the test-retest reliability of EFs tasks [23,25], we must contend with small sample sizes, limiting the scope of generalization of the results. For the factor analysis of TOL and GNG indicators, the subject-to-variable ratio was generally within accepted limits (approximately 10 to 1 [56]), but for the factor analysis of indicators in different TOL trials, the sample was not large enough. Results obtained for smaller samples tend to be less stable and reliable than for larger samples [57]. In both studies, we analyzed results of only five out of eight TOL trials, due to discrepancies in the number of moves in the optimal solutions between test and retest versions for three of eight trials. Further research should use more trials, with systematic manipulation of different aspects of problem structure. We did not investigate the concurrent validity of the tasks in this research. Although convergent and differential validities of this version of the TOL task have been investigated in the context of other EFs tests [26], there is a need for further research on the validity of different TOL and GNG indicators in the context of other planning tasks. Lastly, the TOL version in our research is a conventional test with low ecological validity; further research should investigate concurrent validity of that version and naturalistic planning tasks [58].

## 5. Conclusions

We conducted two studies to verify psychometric properties of commonly used tasks for planning and motor inhibition assessment. Knowledge of reliability over time and factor structure of cognitive tasks is an important aspect of practical application and is needed for adequate assessment. Overall, Study 1 shows that investigated versions of TOL and GNG tasks have satisfactory test-retest reliability coefficients. Nonetheless, ITT should be interpreted cautiously due to the occurrence of practice effects, which strength can vary depending upon the trial difficulty level. Our results are in line with results obtained for various TOL and GNG tasks in similar samples over varying periods [23,24,26,27]. Study 2 shows the factor structure for TOL and GNG tasks with two factors: planning/inhibition and move efficiency. A more detailed factor structure analysis for TOL indicators in each trial shows a six-factor solution where the first factor, named strategic planning, grouped ITT for more difficult trials, while the remaining five factors, named move efficiency, grouped indicators for each trail separately. Similar to other research, TOL indicators were grouped in different factors [8], with planning time loading the same factor with GNG indicators [48].

TOL and GNG tasks are considered to capture planning, and motor inhibition, respectively [59]. However, according to Lezak et al. [3], there are no pure measures of specific executive functions, and all functions, to different degrees, are involved in each task. Our results show that aspects of planning and motor inhibition appear in different ways in both tasks. Practice effects, suggesting strategy use, occur in more difficult TOL trials, but not in GNG and less difficult TOL trials. Planning time in TOL loaded the same factor with indices of GNG, which may suggest that inhibition plays an important role in thinking how to solve the task. On the other hand, inhibition does not appear to be significant for the TOL task’s execution, even though changing the strategy and additional on-line planning may occur during that period. Results of the analysis of factor structure and ANOVA for all trials in the TOL task suggest that level of difficulty (easy vs. difficult trail) may moderate the degree to which the ITT is capturing a specific aspect of executive functions.

Approaches treating executive functions as complex processes and as interrelated aspects are reflected in different theoretical models. Our results can be understood in the light of Diamond’s theory, which assumes a hierarchical structure of executive functions [12]. Inhibition being a more basic function, is involved in higher-level planning. According to Miyake et al. [51], basic functions of inhibition, shifting, and updating are involved in solving complex tasks like TOL. The degree to which specific functions are involved in the task depends upon the chosen strategy of problem-solving, which, according to Miyake et al. [51], can be influenced by the character of an instruction. In our view, the level of difficulty may also influence which strategy is chosen.

Our findings correspond with the general discussion about the interpretation of TOL indicators [7] and suggest that interpretation should be made for different indicators in connection with other methods (i.e., GNG), which gives a better chance for understanding the complexity of executive functions (planning, inhibition, effective performance). It is worth considering those versions of the task, which have proven reliability coefficients and allow for systematic manipulations of problem structure (level of difficulty, i.e., the minimal number of moves to the solution, number of counter-intuitive moves), and calculation of more indicators.

## Figures and Tables

**Table 1:** Survey on studies for test-retest reliability for Tower of London (TOL).

Authors	Number of Participants	Age Range	Intersession Intervals	Version of TOL	Indicators	Correlation (*r*)	Interclass Correlation (ICC)
Schnirman et al. [19]	34	Undergraduate collage students	5–7 weeks	TOL-Revised	Number of trials in optimum solution	0.70	-
Lowe and Rabbitt [20]	162	60–80	4 weeks	TOL from CANTAB battery	Number of trials in optimum solution	0.60	-
					Average number of moves-4-move problem	0.26	-
					Average number of moves-5-move problem	0.47	-
Syväoja et al. [21]	74	11–13	1 year	TOL from CANTAB battery	Number of trials in optimum solution	0.23	-
Welsh et al. [22]	39	Collage students	5–7 weeks	TOL-Revised	Number of trials in optimum solution	0.70	-
Köstering et al. [23]	27	19–26	1 week	TOL-Freiburg version	Accuracy	0.739	ICC (3,1) = 0.734 ICC (2,1) = 0.690
					Initial thinking time	0.405	ICC (3,1) = 0.390 ICC (2,1) = 0.274
					Movement execution time	0.519	ICC (3,1) = 0.475 ICC (2,1) = 0.348
Tunstall et al. [24]	40	21–55	1 month	Four-disc TOL	Total score	0.47	ICC (3,1) = 0.45 ICC (2,1) = 0.45
	21	5–15				0.80	ICC (3,1) = 0.78 ICC (2,1) = 0.70
Lemay et al. [25]	37	52–80	Three sessions (1,2,3) with 2 weeks intervals	Shallice TOL	Total mean moves	1→2 = 0.45 2→3 = 0.34 1→3 = 0.55	ICC (2,1) = 0.30
					Total mean initial thinking time	1→2 = 0.87 2→3 = 0.83 1→3 = 0.82	ICC (2,1) = 0.83
					Total optimal solution	1→2 = 0.47 2→3 = 0.58 1→3 = 0.31	ICC (2,1) = 0.33
					Problems solved	1→2 = 0.18 2→3 = 0.09 1→3 = 0.43	ICC (2,1) = 0.17
Piper et al. [26]	79	18–22	2 weeks	Phillips TOL (from PEBL)	Total moves	0.15	-
					Total time	0.36	-

CANTAB, Cambridge Neuropsychological Test Automated Batteries. PEBL, Psychology Experiment Building Language.

**Table 2:** Survey on studies for test-retest reliability for Go/No Go (GNG).

Authors	Number of Participants	Age Range	Intersession Intervals	Version of GNG	Indicators	Correlation (*r*)	Interclass Correlation (ICC)
Weafer et al. [27]	128	18–30	8 days	GNG learning task	Commission errors	0.65	-
Brunner et al. [28]	26	22–46	6–18 months	Visual GNG	Reaction time	-	ICC (2,1) = 0.86
Langenecker et al. [29]	28	Collage students	3 weeks	Parametric GNG	Reaction time	0.81	-
					Percentage of correct answers	0.73	-
					inhibitions	0.63	-
Kindlon et al. [30]	71 with ADHD	6–16	2–5 months	Passive avoidance learning task	Reaction time	0.72	-
					Correct responses	0.77	-
					Correct non-responses	0.79	-

**Table 3:** Description of the trials in Tower of London (TOL) task in two timepoints (Time 1 and 2).

Time 1					
Number of trial	1	2	3	6	7
Number of minimal moves to the solution	3	5	5	9	9
Number of indirect moves	0	0	1	4	4
Beginning state	B	A	E	E	E
Goal state	E	C	B	E	A
Number of paths to the solution	1	1	1	5	3
**Time 2**					
Number of trial	1	2	3	6	7
Number of minimal moves to the solution	3	5	5	9	9
Number of indirect moves	0	0	1	4	4
Beginning state	D	B	B	A	A
Goal state	B	C	B	B	D
Number of paths to the solution	1	1	1	1	3

**Table 4:** Descriptive statistics, Student’s *t*-test, and Cohen’s *d* for all indicators on Tower of London (TOL) and Go/No Go (GNG) task.

Measure	Time 1			Time 2			*t*	*p*/*p*′	*d*
	*M*	*SD*	*SEM*	*M*	*SD*	*SEM*			
Indicators in TOL:									
Initial Thinking Time (ITT)	27.84	9.69	2.17	16.33	5.01	1.12	6.20	0.000/0.000	1.39
Execution Time (ET)	43.33	14.23	3.18	43.38	10.30	2.30	−0.02	0.986/1.000	-
Full Time (FT)	71.17	21.99	4.92	59.71	13.97	3.12	2.66	0.016/0.080	-
Extra Moves (EM)	3.95/1.12 ^a^	3.78/0.73 ^b^	0.84/0.16 ^c^	3.95/0.94 ^a^	5.98/0.83 ^b^	1.34/0.18 ^c^	0.60	0.555/1.000	-
Indicator in GNG:									
No Go Errors (NGE)	9.50/1.76 ^a^	7.79/0.80 ^b^	1.74/0.18 ^c^	12.05/1.90 ^a^	11.38/0.74 ^b^	2.55/0.16 ^c^	−0.87	0.395/1.000	-
Reaction Time for Go Responses (RTGR)	431.53	74.48	16.65	418.37	74.89	16.75	0.97	0.344/1.000	-

^a^ Mean after Box-Cox transformation. ^b^ Standard deviation after Box-Cox transformation. ^c^ Standard error of measurement after Box-Cox transformation.

**Table 5:** Person’s *r*, interclass correlation (ICC), and reliable change indices (RCI) for all indicators on Tower of London (TOL) and Go/No Go (GNG) task.

Measure	*r*	*p*/*p*′	95% CI	ICC	*p*/*p*′	95% CI	*SED_I_*	95% CI
Indicators in TOL:								
Initial Thinking Time (ITT)	0.52	0.020/0.100	0.18, 0.77	0.42	0.029/0.087	−0.02, 0.72	7.56	100
Execution Time (ET)	0.35	0.128/0.128	−0.15, 0.77	0.34	0.069/0.138	−0.12, 0.67	14.16	95
Full Time (FT)	0.50	0.025/0.100	0.09, 0.78	0.45	0.020/0.080	0.02, 0.74	18.42	95
Extra Moves (EM)	−0.44	0.052/0.114	−0.79, 0.05	−0.44	0.976/0.796	−0.73, 0.00	1.33	95
Indicator in GNG:								
No Go Errors (NGE)	0.56	0.010/0.060	0.28, 0.77	0.56	0.004/0.024	0.17, 0.80	0.72	100
Reaction Time for Go Responses (RTGR)	0.47	0.038/0.114	−0.13, 0.91	0.50	0.009/0.045	0.09, 0.76	78.32	95

**Table 6:** Descriptive statistics of performance on Tower of London (TOL) and Go/No Go (GNG) task in first timepoint.

Measure	Time 1		
	*M*	*SD*	*SEM*
Indicators in TOL:			
Initial Thinking Time (ITT)	28.99/0.96 ^a^	11.12/0.01 ^b^	1.14/0.00 ^c^
Execution Time (ET)	45.02/0.98 ^a^	13.23/0.01 ^b^	1.36/0.00 ^c^
Extra Moves (EM)	4.36/0.88 ^a^	4.29/0.49 ^b^	0.44/0.05 ^c^
Indicator in GNG:			
No Go Errors (NGE)	8.54/1.80 ^a^	6.49/0.63 ^b^	0.66/0.06 ^c^
Reaction Time for Go Responses (RTGR)	441.03/1.00 ^a^	73.09/0.00 ^b^	7.50/0.00 ^c^

^a^ Mean after Box-Cox transformation. ^b^ Standard deviation after Box-Cox transformation. ^c^ Standard error of measurement after Box-Cox transformation.

**Table 7:** Factor loadings of Tower of London (TOL) and Go/No Go (GNG) task.

Measure	Component	
	Factor 1	Factor 2
Extra Moves (EM)		0.899
Initial Thinking Time (ITT)	0.547	
Execution Time (ET)		0.829
No Go Errors (NGE)	−0.830	
Reaction Time for Go Responses (RTGR)	0.788	
Variance (%) explained by each factor	34.93%	31.26%
Cumulative explained variance %	34.93%	66.19%

Factor loadings for all components are presented in Table S5.

**Table 8:** Factor loadings of five trials of Tower of London (TOL).

Measure	Component					
	Factor 1	Factor 2	Factor 3	Factor 4	Factor 5	Factor 6
Trial 1: Extra Moves (EM)		0.860				
Trial 2: Extra Moves (EM)			0.782			
Trial 3: Extra Moves (EM)				0.959		
Trial 6: Extra Moves (EM)					0.936	
Trial 7: Extra Moves (EM)						0.860
Trial 1: Initial Thinking Time (ITT)			0.439			−0.401
Trial 2: Initial Thinking Time (ITT)	0.475	0.509				
Trial 3: Initial Thinking Time (ITT)	0.681					
Trial 6: Initial Thinking Time (ITT)	0.828					
Trial 7: Initial Thinking Time (ITT)	0.760					
Trial 1: Execution Time (ET)		0.920				
Trial 2: Execution Time (ET)			0.850			
Trial 3: Execution Time (ET)				0.938		
Trial 6: Execution Time (ET)					0.835	
Trial 7: Execution Time (ET)			0.438			0.408
Variance (%) explained by each factor	16.34%	14.55%	13.27%	12.61%	11.58%	8.65%
Cumulative explained variance %	16.34%	30.89%	44.16%	56.77%	68.35%	77.00%

Factor loadings for all components are presented in Table S6.

## Data Availability

Data and materials for the experiments reported here are available at https://github.com/psychological-research/tol-and-gng (accessed on 22 September 2021).

## Supplementary Materials

The following are available online at https://www.mdpi.com/article/10.3390/brainsci11111420/s1, Figure S1: Exploratory factor analysis for both tasks showed a 2-factor solution; Figure S2: Exploratory factor analysis for five TOL trials showed a 6-factor solution; Table S1: Performance in Tower of London (TOL) task for eight trials, across repeated measurements: Initial Thinking Time (ITT) and Execution Time (ET); Table S2. Significance of *p* value for three indicators of Tower of London (TOL): post hoc for main effect of “Trial”, and pairwise comparisons for main effect of “Time” and for interaction between “Time” and “Trial”; Table S3. Correlation matrix for Tower of London (TOL) and Go/No Go (GNG) task performance measures; Table S4. Performance in Tower of London (TOL) task for eight trials: Initial Thinking Time (ITT), Execution Time (ET), and Extra Moves (EM); Table S5. Factor loadings of Tower of London (TOL) and Go/No Go (GNG) task; Table S6. Factor loadings of five trials of Tower of London (TOL).

## References

[1] Chan R.C., Shum D., Toulopoulou T., Chen E.Y. (2008). Assessment of executive functions: Review of instruments and identification of critical issues. Arch. Clin. Neuropsychol..

[2] Alvarez J.A., Emory E. (2006). Executive function and the frontal lobes: A meta-analytic review. Neuropsychol. Rev..

[3] Lezak M.D., Howieson D.B., Loring D.W. (2004). Neuropsychological Assessment.

[4] Grafman J., Litvan I. (1999). Importance of deficits in executive functions. Lancet.

[5] Berg K.W., Byrd D.L. (2002). The Tower of London spatial problem-solving task: Enhancing clinical and research implementation. J. Clin. Exp. Neuropsychol..

[6] Sullivan J.R., Riccio C.A., Castillo C.L. (2009). Concurrent validity of the tower tasks as measures of executive function in adults: A meta-analysis. Appl. Neuropsychol..

[7] Kaller C.P., Rahm B., Köstering L., Unterrainer J.M. (2011). Reviewing the impact of problem structure on planning: A software tool for analyzing tower tasks. Behav. Brain Res..

[8] Berg W.K., Byrd D.L., McNamara J.P., Case K. (2010). Deconstructing the tower: Parameters and predictors of problem difficulty on the Tower of London task. Brain Cogn..

[9] Ouellet M.C., Beauchamp M.H., Owen A.M., Doyon J. (2004). Acquiring a cognitive skill with a new repeating version of the Tower of London task. Can. J. Exp. Psychol..

[10] Bari A., Robbins T.W. (2013). Inhibition and impulsivity: Behavioral and neural basis of response control. Prog. Neurobiol..

[11] Mirabella G. (2021). Inhibitory control and impulsive responses in neurodevelopmental disorders. Dev. Med. Child. Neurol..

[12] Diamond A. (2013). Executive functions. Annu. Rev. Psychol..

[13] Nigg J.T. (2000). On inhibition/disinhibition in developmental psychopathology: Views from cognitive and personality psychology and a working inhibition taxonomy. Psychol. Bull..

[14] Raud L., Westerhausen R., Dooley N., Huster R.J. (2020). Differences in unity: The go/no-go and stop signal tasks rely on different mechanisms. Neuroimage.

[15] Bezdjian S., Baker L.A., Lozano D.I., Raine A. (2009). Assessing inattention and impulsivity in children during the Go/ NoGo task. Br. J. Dev. Psychol..

[16] Calamia M., Markon K., Tranel D. (2013). The robust reliability of neuropsychological measures: Meta-analyses of test-retest correlations. Clin. Neuropsychol..

[17] Duff K. (2012). Evidence-based indicators of neuropsychological change in the individual patient: Relevant concepts and methods. Arch. Clin. Neuropsychol..

[18] Koo T.K., Li M.Y. (2016). A guideline of selecting and reporting Intraclass Correlation Coefficients for reliability research. J. Chiropr. Med..

[19] Schnirman G.M., Welsh M.C., Retzlaff P.D. (1998). Development of the Tower of London-revised. Assessment.

[20] Lowe C., Rabbitt P. (1998). Test\re-test reliability of the CANTAB and ISPOCD neuropsychological batteries: Theoretical and practical issues. Neuropsychologia.

[21] Syväoja H.J., Tammelin T.H., Ahonen T., Räsänen P., Tolvanen A., Kankaanpää A., Kantomaa M.T. (2015). Internal consistency and stability of the CANTAB neuropsychological test battery in children. Psychol. Assess..

[22] Welsh M.C., Revilla V., Strongin D., Kepler M. (2000). Towers of Hanoi and London: Is the nonshared variance due to differences in task administration?. Percept. Mot. Skills.

[23] Köstering L., Nitschke K., Schumacher F.K., Weiller C., Kaller C.P. (2015). Test-retest reliability of the Tower of London Planning Task (TOL-F). Psychol. Assess..

[24] Tunstall J.R., O’Gorman J.G., Shum D.H. (2016). A four-disc version of the Tower of London for clinical use. J. Neuropsychol..

[25] Lemay S., Bédard M.A., Rouleau I., Tremblay P.L. (2004). Practice effect and test-retest reliability of attentional and executive tests in middle-aged to elderly subjects. Clin. Neuropsychol..

[26] Piper B.J., Mueller S.T., Geerken A.R., Dixon K.L., Kroliczak G., Olsen R.H., Miller J.K. (2015). Reliability and validity of neurobehavioral function on the Psychology Experimental Building Language test battery in young adults. PeerJ.

[27] Weafer J., Baggott M.J., de Wit H. (2013). Test-retest reliability of behavioral measures of impulsive choice, impulsive action, and inattention. Exp. Clin. Psychopharmacol..

[28] Brunner J.F., Hansen T.I., Olsen A., Skandsen T., Håberg A., Kropotov J. (2013). Long-term test-retest reliability of the P3 NoGo wave and two independent components decomposed from the P3 NoGo wave in a visual Go/NoGo task. Int. J. Psychophysiol..

[29] Langenecker S., Zubieta J.K., Young E., Akil H., Nielson K. (2007). A task to manipulate attentional load, set-shifting, and inhibitory control: Convergent validity and test-retest reliability of the Parametric Go/No-Go Test. J. Clin. Exp. Neuropsychol..

[30] Kindlon D.J., Mezzacappa E., Earls F. (1995). Psychometric properties of impulsivity measures: Temporal stability, validity, and factor structure. J. Child. Psychol. Psychiatry.

[31] Shallice T. (1982). Specific impairments of planning. Philos. Trans. R Soc. Lond. B Biol. Sci..

[32] Frydecka D., Małyszczak K., Chachaj A., Kiejna A. (2010). Factorial structure of the general health questionnaire (GHQ-30). Psychiatr. Pol..

[33] Mueller S.T., Piper B.J. (2014). The Psychology Experiment Building Language (PEBL) and PEBL Test Battery. J. Neurosci. Methods.

[34] Ward G., Allport A. (1997). Planning and problem-solving using the five-disc Tower of London task. Q J. Exp. Psychol. B.

[35] Phillips L.H., Wynn V., Gilhooly K.J., Della Sala S., Logie R.H. (1999). The role of memory in the Tower of London task. Memory.

[36] Sakia R.M. (1992). The Box-Cox transformation technique: A review. J. R. Stat. Soc..

[37] Schatz P., Ferris C.S. (2013). One-month test-retest reliability of the ImPACT Test Battery. Arch. Clin. Neuropsychol..

[38] Weir J.P. (2005). Quantifying test-retest reliability using the intraclass correlation coefficient and the SEM. J. Strength Cond Res..

[39] Holm S. (1979). A simple sequentially rejective multiple test procedure. Scand. J. Statist..

[40] Jacobson N.S., Truax P. (1992). Clinical significance: A statistical approach to defining meaningful change in psychotherapy research. J. Consult Clin. Psychol..

[41] Iverson G.L., Lovell M.R., Collins M.W. (2003). Interpreting change on ImPACT following sport concussion. Clin. Neuropsychol..

[42] Chelune G.J., Naugle R.I., Luders H., Sedlak J., Awad I.A. (1993). Individual change after epilepsy surgery: Practice effects and base-rate information. Neuropsychology.

[43] Iverson G.L. (2001). Interpreting change on the WAIS-III/WMS-III in clinical samples. Arch. Clin. Neuropsychol..

[44] Beglinger L.J., Gaydos B., Tangphao-Daniels O., Duff K., Kareken D.A., Crawford J., Fastenau P.S., Siemers E.R. (2005). Practice effects and the use of alternate forms in serial neuropsychological testing. Arch. Clin. Neuropsychol..

[45] Mirabella G. (2018). The weight of emotions in decision-making: How fearful and happy facial stimuli modulate action readiness of goal-directed actions. Front. Psychol..

[46] Mancini C., Falciati L., Maioli C., Mirabella G. (2020). Threatening Facial Expressions Impact Goal-Directed Actions Only if Task-Relevant. Brain Sci..

[47] Spadacenta S., Gallese V., Fragola M., Mirabella G. (2014). Modulation of arm reaching movements during processing of arm/hand-related action verbs with and without emotional connotation. PLoS ONE.

[48] Levin H.S., Fletcher J.M., Kufera J.A., Harward H., Lilly M.A., Mendelsohn D., Bruce D., Eisenberg H.M. (1996). Dimensions of cognition measured by the Tower of London and other cognitive tasks in head-injured children and adolescents. Dev. Neuropsychol..

[49] Culbertson W.C., Zillmer E.A. (1998). The construct validity of the Tower of London DX as a measure of the executive functioning of ADHD children. Assessment.

[50] Georgiou G.K., Li J., Das J.P. (2017). Tower of London: What Level of Planning Does it Measure?. Psychol. Stud..

[51] Miyake A., Friedman N.P., Emerson M.J., Witzki A.H., Howerter A., Wager T.D. (2000). The unity and diversity of executive functions and their contributions to complex “frontal lobe” tasks: A latent variable analysis. Cogn. Psychol..

[52] Bender A.D., Filmer H.L., Garner K.G., Naughtin C.K., Dux P.E. (2016). On the relationship between response selection and response inhibition: An individual differences approach. Atten. Percept. Psychophys.

[53] Snodgrass J.G., Luce R.D., Galanter E. (1967). Some Experiments on Simple and Choice Reaction Time. J. Exp. Psychol..

[54] Kafer K.L., Hunter M. (1997). On testing the face validity of planning/problem-solving tasks in a normal population. J. Int. Neuropsychol. Soc..

[55] Köstering L., Schmidt C.S., Egger K., Amtage F., Peter J., Klöppel S., Beume L., Hoeren M., Weiller C., Kaller C.P. (2015). Assessment of planning performance in clinical samples: Reliability and validity of the Tower of London task (TOL-F). Neuropsychologia.

[56] Tabachnick B.G., Fidell L.S. (1989). Using Multivariate Statistics.

[57] Gorsuch R.L. (1983). Factor Analysis.

[58] Greenwood K.E., Wykes T., Sigmundsson T., Landau S., Morris R.G. (2011). Tower of London versus real life analogue planning in schizophrenia with disorganization and psychomotor poverty symptoms. J. Int. Neuropsychol. Soc..

[59] Jurado M.B., Rosselli M. (2007). The elusive nature of executive functions: A review of our current understanding. Neuropsychol. Rev..

