# Supplementary material for: Assessment of Motor Planning and Inhibition Performance in Non-Clinical Sample—Reliability and Factor Structure of the Tower of London and Go/No Go Computerized Tasks
Source: Brain Sci. 2021 Oct 27;11(11):1420. doi: 10.3390/brainsci11111420 (PMC8615804; doi:10.3390/brainsci11111420)
Supplement: Supplementary file 1 [file brainsci-11-01420-s001.zip › brainsci-1411499-supplementary.pdf]

Supplementary Dates

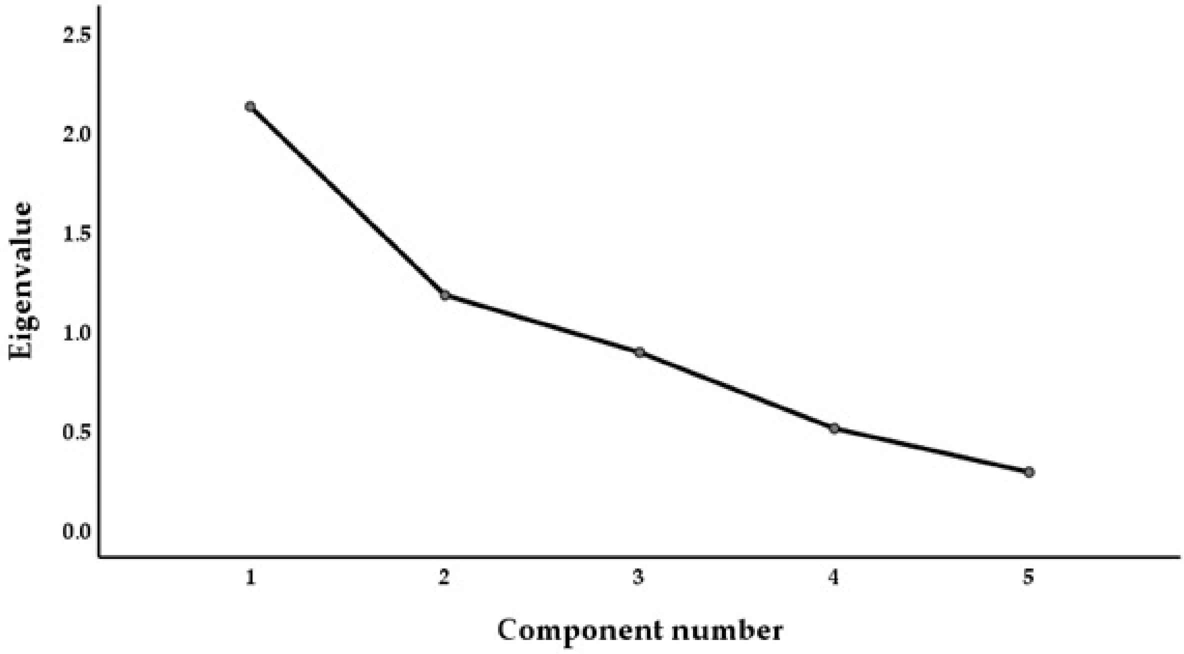

Figure S1. Exploratory factor analysis for both tasks showed a 2-factor solution.

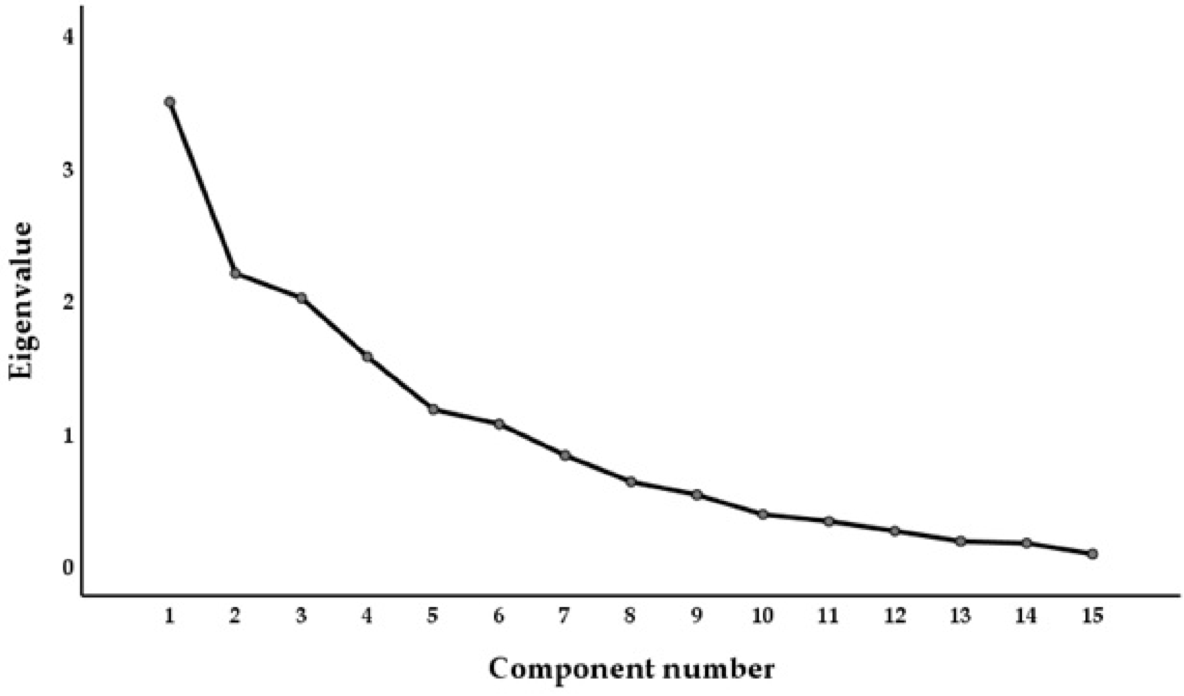

Figure S2. Exploratory factor analysis for five TOL trials showed a 6-factor solution.

Table S1. Performance in Tower of London (TOL) task for eight trials, across repeated measurements: Initial Thinking Time (ITT) and Execution Time (ET).

Measure	Time 1			Time 2		
	M	*SD*	*SEM*	*M*	*SD*	*SEM*
Initial Thinking Time (ITT)						
Trial 1	3.53	1.23	0.27	3.99	1.23	0.28
Trial 2	2.64	0.63	0.14	3.15	1.47	0.33
Trial 3	7.16	3.49	0.78	2.92	1.36	0.30
Trial 4	8.77	4.62	1.03	2.96	1.44	0.32
Trial 5	3.84	1.23	0.28	2.62	0.99	0.22
Trial 6	7.47	3.69	0.83	3.89	1.60	0.36
Trial 7	7.04	4.48	1.00	2.38	0.69	0.15
Trial 8	4.80	2.73	0.61	4.82	1.75	0.39
Execution Time (ET)						
Trial 1	3.54	3.33	0.75	4.00	2.91	0.65
Trial 2	5.10	1.54	0.34	6.72	3.37	0.75
Trial 3	8.35	5.18	1.16	6.26	2.40	0.54
Trial 4	11.45	4.79	1.07	12.30	7.64	1.71
Trial 5	3.64	1.74	0.39	8.97	2.51	0.56
Trial 6	15.34	8.58	1.92	13.51	4.76	1.06
Trial 7	10.99	3.25	0.73	12.89	4.35	0.97
Trial 8	15.68	8.31	1.86	17.07	6.72	1.50

Table S2. Significance of p value for three indicators of Tower of London (TOL): post hoc for main effect of "Trial", and pairwise comparisons for main effect of "Time" and for interaction between "Time" and "Trial".

Significance of *p* value for Initial Thinking Time (ITT)				
Post hoc for main effect of "Trial"				
	Trial 1	Trial 2	Trial 3	Trial 6
Trial 2	0.000			
Trial 3	0.467	0.000		
Trial 6	0.004	0.000	0.637	
Trial 7	1.000	0.003	0.821	0.038
Pairwise comparisons for main effect of "Time"				
Time 1	Time 2	Trial 1	0.135	
Time 1	Time 2	Trial 2	0.142	
Time 1	Time 2	Trial 3	0.000	
Time 1	Time 2	Trial 6	0.000	
Time 1	Time 2	Trial 7	0.000	
Pairwise comparisons for interaction effect between "Time" and "Trial"				
Time 1				
	Trial 1	Trial 2	Trial 3	Trial 6
Trial 2	0.001			
Trial 3	0.000	0.000		
Trial 6	0.000	0.000	1.000	
Trial 7	0.002	0.000	1.000	1.000
Time 2				
	Trial 1	Trial 2	Trial 3	Trail 6
Trial 2	0.009			
Trial 3	0.001	1.000		
Trial 6	1.000	0.563	0.036	
Trial 7	0.000	0.011	0.601	0.008
Significance of *p* value for Execution Time (ET)				
Post hoc for main effect of "Trial"				
	Trial 1	Trial 2	Trial 3	Trial 6
Trial 2	0.000			
Trial 3	0.000	0.494		
Trial 6	0.000	0.000	0.000	
Trial 7	0.000	0.000	0.000	0.176

Table S3. Correlation matrix for Tower of London (TOL) and Go/No Go (GNG) task performance measures.

	Initial Thinking Time (ITT)	Execution Time (ET)	No Go Errors (NGE)	Reaction Time for Go Responses (RTGR)
Extra Moves (EM)	0.03	0.56***	-0.10	0.09
Initial Thinking Time (ITT)		0.04***	-0.28**	0.17
Execution Time (ET)			-0.27*	0.30**
No Go Errors (NGE)				-0.49***

* $p < 0.05$. ** $p < 0.01$. *** $p < 0.001$.

Table S4. Performance in Tower of London (TOL) task for eight trials: Initial Thinking Time (ITT), Execution Time (ET), and Extra Moves (EM).

Measure	*M*	*SD*	*SEM*
Initial Thinking Time (ITT)			
Trial 1	3.82	1.41	0.14
Trial 2	3.01	1.30	0.13
Trial 3	7.39	3.48	0.36
Trial 4	7.88	4.34	0.45
Trial 5	4.42	2.02	0.21
Trial 6	7.42	4.37	0.45
Trial 7	7.35	5.06	0.52
Trial 8	4.78	2.25	0.23
Execution Time (ET)			
Trial 1	3.49	2.35	0.24
Trial 2	5.99	3.29	0.34
Trial 3	8.50	6.57	0.67
Trial 4	14.69	7.18	0.74
Trial 5	4.11	2.06	0.21
Trial 6	15.95	6.45	0.66
Trial 7	11.09	3.11	0.32
Trial 8	14.58	6.03	0.62
Extra Moves (EM)			
Trial 1	3.49	2.35	0.24
Trial 2	5.99	3.29	0.34
Trial 3	8.50	6.57	0.67
Trial 4	14.69	7.18	0.74
Trial 5	4.11	2.06	0.21
Trial 6	15.95	6.45	0.66
Trial 7	11.09	3.11	0.32
Trial 8	14.58	6.03	0.62

Table S5. Factor loadings of Tower of London (TOL) and Go/No Go (GNG) task.

Measure	Component	
	Factor 1	Factor 2
Extra Moves (EM)	-0.050	0.899
Initial Thinking Time (ITT)	0.547	0.253
Execution Time (ET)	0.368	0.829
No Go Errors (NGE)	-0.830	-0.035
Reaction Time for Go Responses (RTGR)	0.788	0.048
Variance (%) explained by each factor	34.93%	31.26%
Cumulative explained variance %	34.93%	66.19%

Table S6. Factor loadings of five trials of Tower of London (TOL).

Measure	Component					
	Factor 1	Factor 2	Factor 3	Factor 4	Factor 5	Factor 6
Trial 1: Extra Moves (EM)	-0.184	0.860	-0.113	-0.054	-0.088	0.047
Trial 2: Extra Moves (EM)	-0.233	-0.193	0.782	0.027	0.124	0.068
Trial 3: Extra Moves (EM)	-0.089	-0.071	0.001	0.959	0.051	-0.007
Trial 6: Extra Moves (EM)	-0.090	-0.078	0.010	0.097	0.936	0.081
Trial 7: Extra Moves (EM)	0.101	-0.031	0.010	-0.052	0.073	0.860
Trial 1: Initial Thinking Time (ITT)	0.341	0.382	0.439	0.066	0.148	-0.401
Trial 2: Initial Thinking Time (ITT)	0.475	0.509	0.342	0.110	-0.051	-0.221
Trial 3: Initial Thinking Time (ITT)	0.681	0.153	0.026	0.078	0.214	-0.305
Trial 6: Initial Thinking Time (ITT)	0.828	-0.125	-0.149	0.027	0.040	0.156
Trial 7: Initial Thinking Time (ITT)	0.760	-0.047	0.070	-0.121	-0.012	0.197
Trial 1: Execution Time (ET)	0.072	0.920	0.137	0.052	0.060	-0.037
Trial 2: Execution Time (ET)	0.099	0.208	0.850	0.167	0.045	-0.046
Trial 3: Execution Time (ET)	0.072	0.108	0.174	0.938	0.059	-0.097
Trial 6: Execution Time (ET)	0.347	0.061	0.256	0.014	0.835	0.008
Trial 7: Execution Time (ET)	0.357	0.204	0.438	-0.091	0.223	0.408
Variance (%) explained by each factor	16.34%	14.55%	13.27%	12.61%	11.58%	8.65%
Cumulative explained variance %	16.34%	30.89%	44.16%	56.77%	68.35%	77.00%
